# Cone-beam computed tomography prescription by Brazilian orthodontists

**DOI:** 10.1590/2177-6709.30.2.e252486.oar

**Published:** 2025-05-23

**Authors:** Karine EVANGELISTA, Grasielle Di Manoel CAIADO, Maria do Carmo Matias FREIRE, José VALLADARES-NETO, Maria Alves Garcia SILVA

**Affiliations:** 1) Universidade Federal de Goiás, Dental School, Department of Orthodontics (Goiânia/GO, Brazil).; 2) Private practice in Orthodontics (Brazil).; 3) Universidade Federal de Goiás, Dental School, Department of Public Health (Goiânia/GO, Brazil).; 4) Universidade Federal de Goiás, Dental School, Department of Stomatology (Goiânia/GO, Brazil).

**Keywords:** Cone-beam computed tomography, Orthodontics, Prescription, Tomografia computadorizada de feixe cônico, Ortodontia, Prescrição

## Abstract

**Objective:**

To investigate the factors that influence Cone-beam computed tomography (CBCT) imaging prescription in Brazilian orthodontics practice.

**Methods:**

This cross-sectional study was performed in 2020 using an online survey sent to all Brazilian orthodontists registered at the Federal Council of Dentistry. The variables were the orthodontists’ demographic features and the CBCT prescription in clinical practice. A descriptive and comparative analysis was implemented using frequencies and the chi-square test, respectively.

**Results:**

The sample consisted of 939 respondents. CBCT prescription and the use of guidelines for imaging in orthodontics were confirmed by 81.9% and 52.6% of the sample, respectively. Training for CBCT was reported by 37.0%, mainly during specialization programs in orthodontics (50.0%), about one to five years ago (64.7%), with a duration of 4 to 8 hours (53.4%). The CBCT prescription was indicated predominantly for specific cases (71.0%), and was generally related to impacted teeth (74.7%), orthognathic surgical patients (46.2%), and root resorption (41.9%). The results indicated differences in CBCT prescription between the Brazilian macro-regions, regarding the criteria for prescription, use of guidelines, reasons for not recommending the exam, diagnostic purposes, voxel size, and training for the use of CBCT (p<0.05).

**Conclusion:**

The prescription of CBCT was adequate by most Brazilian orthodontists for cases involving impacted teeth, surgical interventions, and root resorption. However, adherence to imaging prescription guidelines was observed in less than 50% of the sample. There is a deficiency in understanding technical parameters and a lack of specific training on CBCT. Disparities were evident among the Brazilian macro-regions, particularly concerning CBCT costs and other variables analyzed.

## RESUMO

**Objetivo:**

Investigar os fatores que influenciam a prescrição de exames de tomografia computadorizada de feixe cônico (TCFC) na prática ortodôntica brasileira.

**Métodos:**

Este estudo transversal foi realizado em 2020, por meio de uma pesquisa online enviada a todos os ortodontistas brasileiros registrados no Conselho Federal de Odontologia. As variáveis foram as características demográficas dos ortodontistas e a prescrição de TCFC na prática clínica. Análises descritiva e comparativa foram implementadas por meio de frequências e do teste qui-quadrado, respectivamente.

**Resultados:**

A amostra consistiu em 939 respondentes. A prescrição de TCFC e o uso de diretrizes para prescrição de imagens em Ortodontia foram confirmados por 81,9% e 52,6% da amostra, respectivamente. O treinamento para TCFC foi relatado por 37,0%, principalmente durante programas de especialização em Ortodontia (50,0%), feitos há cerca de um a cinco anos atrás (64,7%), com duração de 4 a 8 horas (53,4%). A prescrição de TCFC foi indicada predominantemente para casos específicos (71,0%), geralmente relacionada a dentes impactados (74,7%), pacientes submetidos a cirurgia ortognática (46,2%) e reabsorção radicular (41,9%). Os resultados indicaram diferenças na prescrição de TCFC entre as regiões brasileiras, com relação aos critérios de prescrição, seguimento de diretrizes, razões para não recomendar o exame, propósitos diagnósticos, tamanho de *voxel* e treinamento para o uso de TCFC (p < 0,05).

**Conclusão:**

A prescrição de TCFC foi adequada por parte dos ortodontistas brasileiros para casos envolvendo dentes impactados, intervenções cirúrgicas e reabsorção radicular. No entanto, a adesão às diretrizes para prescrição de exames de imagem foi observada em aproximadamente50% da amostra. Há uma deficiência no entendimento dos parâmetros técnicos e uma falta de treinamento específico em TCFC. Disparidades entre as regiões brasileiras foram evidentes, particularmente em relação aos custos de TCFC e outras variáveis analisadas.

## INTRODUCTION

In Orthodontics, the conventional method of collecting data for the diagnosis and treatment planning of malocclusions typically involves combining two-dimensional (2D) radiographs (such as lateral cephalometry, panoramic, and periapical radiographs) with clinical examinations and analyses conducted on either plaster or digital models. However, 2D images have inherent limitations concerning anatomical overlap and distortion. Consequently, the use of Cone-beam computed tomography (CBCT) has become increasingly common in orthodontic practice, offering significant contributions to the diagnosis and treatment planning in both adult and pediatric patients.^1^

However, it is crucial to note that the routine and, at times, unjustified use of tomographic examinations has raised serious concerns in Orthodontics.^1^ ^,^ ^2^ In line with this, guidelines for imaging prescription have been developed based on radiation dose, to ensure the appropriate use of CBCT in Orthodontics. It is paramount for professionals to initially adhere to the ALARA principle (As Low as Reasonably Achievable), particularly given that CBCT is associated with the highest dose of ionizing radiation.^1^ ^-^ ^4^ Strict adherence to this principle becomes significantly important due to the increasing demand for orthodontic treatment among young patients, who are more vulnerable to the effects of ionizing radiation.^5^

CBCT is not only about minimizing the radiation dose. Other principles, such as ALADA (As Low as Diagnostically Acceptable)^6^ and ALADAIP (As Low as Diagnostically Acceptable being Indication-oriented and Patient-specific)^7^ play crucial roles. These principles advocate maintaining radiation doses at acceptable levels for diagnosis, while tailoring them to the specific needs of the indication and patient characteristics. This comprehensive approach ensures the judicious use of CBCT in clinical practice.

Although Brazilian regulations do not provide specific training for CBCT interpretation, the meticulous prescription of tomographic images guided by the ALARA, ALADA, and ALADAIP principles requires specific training for diagnostic imaging professionals. In addition to grasping the three-dimensional interpretation of craniofacial morphology, technical knowledge concerning aspects related to the generation of tomographic images is imperative for professionals, to ensure the judicious use of ionizing radiation. The parameters influencing the dose of ionizing radiation received by the patient include: peak kilovoltage (kVp), milliamperage (mA), voxel size, and field of view (FOV) size. These parameters can be adjusted for a low-dose protocol, according to diagnostic purposes.^7^ ^,^ ^8^

In this context, orthodontics specialty programs must incorporate specific training in CBCT usage, to optimize the technique and enhance the practice of image prescription. In Brazil, evidence of these aspects is lacking. Investigating these across the five geographic macro-regions of the country can yield relevant data to support strategies aimed at the rational utilization of 3D images. The present study aimed to explore the aspects associated with the prescription of CBCT images in the clinical practice of orthodontists in Brazil and to ascertain whether discrepancies exist among the five macro-regions of the country.

## MATERIAL AND METHODS

This cross-sectional study analyzed partial data from an international multicenter study that aimed to describe and compare CBCT imaging prescriptions in clinical practice among orthodontists from Belgium, Brazil, Canada, Romania, and the United States.^9^ The project was approved by the Research Ethics Committee of the *Universidade Federal de Goiás*, Brazil (CAAE number: 15742619.7.0000.5083).

The population for this study comprised 23,872 Brazilian dentists registered as orthodontic specialists at the Federal Council of Dentistry on May 27, 2020. The sample size was calculated using simple random and stratified sampling, with proportional allocation methods implemented using R software, version 3.6.2. An acceptable sampling error of 3% with a 95% confidence interval was determined. Based on these criteria, 570 participants were required. To ensure a satisfactory response rate, invitations to participate were extended to all registered orthodontic specialists.

Data were collected using a pretested self-administered electronic questionnaire specifically developed for this study (Supplement 1). From February to September 2020, questionnaires and informed consent forms were emailed to orthodontists using the SurveyMonkey platform. As a standard procedure, reminders were sent one week and one month after the initial distribution of the questionnaires, to increase the participation of the study population.

The variables selected for the present analysis were the demographic and work-related characteristics of the specialists, including sex, age, length of experience, Brazilian macro-region where they practiced as orthodontists, and aspects related to their use of CBCT. These aspects included indication criteria, reasons for not requesting the examination, adherence to guidelines, technical parameters, and training. All variables were treated as categorical variables, and their distributions were described as absolute numbers and percentages (n and %).

Bivariate comparisons were conducted between each variable concerning the prescription of CBCT and the five geographic macro-regions (North, Northeast, Midwest, Southeast, and South) of the respondents’ professional activities using Pearson’s chi-square test, with a significance level set at p < 0.05. Due to the number of respondents in each region, the test considered the expected counts. A *post-hoc* Bonferroni test with adjusted residuals was performed to check for differences among the macro-regions, with a significance level set at ± 2.80. Individuals who did not respond to the questions were excluded from the analysis. Statistical analyses were performed using the Statistical Package for the Social Sciences (SPSS), version 25.0.

## RESULTS

Of the 23,872 Brazilian orthodontists invited to participate in the study, 939 completed the questionnaire, yielding a response rate of 4.2% (Table 1). Most respondents were male, aged between 20 and 49, with six or more years of experience working as specialists in Orthodontics and practicing the specialty in the Southeast and South regions.

**Table 1:** Characteristics of orthodontic specialists in Brazil (n= 939).

Variable	n (%)
Sex	
Female	420 (44.7)
Male	514 (54.7)
Not answered	5 (0.5)
Age	
20-39	358 (38.1)
40-49	304 (32.4)
50-59	193 (20.6)
>60	80 (8.5)
Not answered	4 (0.4)
Brazilian macro-regions	
North	63 (6.7)
North East	111 (11.8)
Midwest	150 (6.0)
South	190 (20.2)
South East	402 (42.8)
Not answered	23 (2.4)
Number of years as a licensed orthodontist	
< 5	196 (20.9)
6-20	453 (48.2)
> 20	290 (30.9)

Of the total sample (n = 939), 81.9% indicated that they had used CBCT in clinical practice (Table 2). Tomographic evaluations were primarily based on images of tomographic sections (46.1%) or combined with 3D image models (52.7%). A minority of professionals reported having a tomography machine in their office (2.4%), while the majority prescribed tomography scans for selected cases (71.0%).

**Table 2:** Prescription of CBCT imaging and adherence to guidelines by orthodontists in Brazil, stratified by geographic macro-regions (n=939).

Variables	Total	Brazilian macro-regions					Adjusted residual	p-value
		North	North East	Midwest	South	South East		
	n/%	n/%	n/%	n/%	n/%	n/%		
Prescribe CBCT								
Yes	747 (81.9)	46 (74.2)	94 (84.7)	122 (82.4)	145 (76.3)	340 (84.8)	-2.3 (2.0)	0.053
No	165 (18.1)	16 (25.8)	17 (15.3)	26 (17.6)	45 (23.7)	61 (15.2)		
Not answered	27							
How do you use CBCT images?								
To evaluate all axial, coronal and sagittal slices	340 (46.1)	24 (52.2)	36 (39.6)	61 (50.0)	69 (48.6)	150 (44.5)	-1.3 (1.0)	0.449
For 3D model analysis	9 (1.2)	1 (2.2)	2 (2.2)	2 (1.6)	2 (1.4)	2 (0.6)	-1.4 (0.9)	0.665
Both	389 (52.7)	21 (45.7)	53 (58.2)	59 (48.8)	71 (50.0)	185 (54.9)	-1.1 (1.1)	0.402
Not answered	201							
Have a CBCT machine in your own office?								
Yes	18 (2.4)	1 (2.2)	0 (0)	2 (1.6)	2 (1.4)	13 (3.8)	-1.6 (2.3)	0.195
No	722 (97.6)	45 (97.8)	92 (100)	120 (98.4)	139 (98.6)	326 (96.2)		
Not answered	199							
Criteria used for prescribing CBCT in orthodontic practice*								
For treatment planning purposes, in every case	9 (1.0)	0 (0.0)	1 (0.9)	0 (0.0)	0 (0.0)	8 (3.9)	-1.3 (2.7)	0.017
For treatment planning purposes, in most of cases	55 (6.0)	4 (6.3)	3 (2.7)	8 (5.3)	9 (4.7)	31 (7.7)	-1.6 (1.9)	0.043
During treatment, in every case	9 (1.0)	0 (0.0)	0 (0.0)	1 (0.7)	0 (0.0)	8 (2.0)	-1.5 (2.7)	0.018
After treatment, in every case	8 (0.9)	1 (1.6)	1 (0.9)	0 (0.0)	1 (0.5)	5 (1.2)	-1.3 (1.1)	0.089
For selected cases only	650 (71.0)	36 (57.1)	86 (77.5)	110 (73.3)	128 (67.4)	290 (72.1)	-2.5 (1.6)	0.057
Not answered	213							
Follow guidelines								
Yes	387 (52.6)	20 (43.5)	43 (46.7)	60 (49.2)	69 (48.6)	195 (58.4)	-1.3 (2.9)	0.067
No	349 (47.4)	26 (56.5)	49 (53.3)	62 (50.8)	73 (51.4)	139 (41.6)		
Not answered	203							
Guideline followed*								
AAROM	170 (18.6)	8 (12.7)	20 (18.0)	21 (14.0)	31 (16.3)	90 (22.4)	-1.6 (2.6)	0.004
SEDENTEXCT	23 (2.5)	0 (0.0)	0 (0.0)	2 (1.3)	7 (3.7)	14 (3.5)	-1.3 (1.7)	0.001
Local or national	206 (22.5)	9 (14.3)	24 (21.6)	38 (25.3)	33 (17.4)	102 (25.4)	-1.6 (1.8)	0.005
Other	357 (39)	16 (25.4)	40 (36)	57 (38.0)	60 (31.6)	184 (45.8)	-2.3 (2.7)	0.476
Not answered	542							
Reason for not prescribing CBCT**								
High cost	436 (47.3)	35 (55.6)	57 (51.4)	78 (52.0)	80 (42.1)	186 (46.3)	-1.7 (1.3)	0.002
Not having a specialized radiology center nearby	33 (3.6)	4 (6.3)	7 (6.3)	4 (2.7)	7 (3.7)	11 (2.7)	-1.2 (1.2)	0.025
CBCT will bring no new information	15 (1.6)	1 (1.6)	1 (0.9)	2 (1.3)	4 (2.1)	7 (1.7)	-0.7 (0.6)	0.117
Not answered	232							

*Total of respondents who answered “yes” to CBCT prescription and to follow guidelines. **Total of respondents who answered “no” to CBCT prescription. Pearson’s Chi-square test: Statistically significant results for bivariate analysis (p<0.05). Bonferroni *post-hoc* test: values less than ± 2.80 of the adjusted residuals do not indicate statistical differences between the macro-regions.

Among all the respondents, 52.6% reported following guidelines for CBCT prescription, with a higher utilization of local guidelines (22.5%), followed by the American Association of Oral and Maxillofacial Radiology (AAOMR) guidelines (18.6%) (Table 2). The primary reason for not prescribing CBCT was its high cost (47.3%), particularly among orthodontists in the North (55.6%), Midwest (52.0%), and Northeast (51.4 %) regions, with a significant p-value of 0.002. Significant differences were also observed regarding the second reason reported by the respondents, not having a specialized radiology center nearby, which was more frequent in the North (6.3%) and Northeast (6.3%) regions, with a p-value of 0.025.

The diagnostic purposes for prescribing CBCT and technical parameters are detailed in Tables 3 and 4, respectively. Among the specialists recommending this imaging examination, the most common criterion was impacted teeth (74.7%), followed by surgical cases (46.2%) and root resorption (41.9%). Clinical prescriptions related to cleft palate accounted for 22.5% of the total prescriptions. When comparing Brazilian regions, the North region (60.3%) exhibited a lower frequency of prescriptions for impacted teeth (p = 0.046). The Midwest region (50.7%) indicated that CBCT was most frequently used for root resorption analysis (p = 0.001).

**Table 3:** Diagnostic purposes for CBCT prescriptions by orthodontists in Brazil, stratified by geographic macro-regions (n=916^**^).

Variables	Total	Brazilian macro-regions				Adjusted residual		p-value
		North	North East	Midwest	South	South East		
	n (%)	n (%)	n (%)	n (%)	n (%)	n (%)		
Impacted teeth	684 (74.7)	38 (60.3)	87 (78.4)	118 (78.7)	132 (69.5)	309 (76.9)	-2.7 (1.3)	0.046
Root resorption	384 (41.9)	22 (34.9)^A.B^	55 (49.5)^A.B^	76 (50.7)^B^	81 (42.6)^A.B^	150 (37.3)^A^	-1.9 (3.8)	0.001*
Dental anomalies	296 (32.3)	15 (23.8)	36 (32.4)	52 (34.7)	59 (31.1)	134 (33.3)	-1.5 (0.7)	0.168
Facial asymmetry	291 (31.8)	14 (22.2)	37 (33.3)	54 (36.0)	55 (28.9)	131 (32.6)	-1.7 (1.2)	0.132
Dental position	241 (26.3)	12 (19.0)	29 (26.1)	39 (26.0)	43 (22.6)	118 (29.4)	-1.4 (1.8)	0.105
Upper airway analysis	144 (15.7)	7 (11.1)	20 (18.0)	23 (15.3)	25 (13.2)	69 (17.2)	-1.1 (1.1)	0.147
Palate cleft	206 (22.5)	10 (15.9)	24 (21.6)	39 (26.0)	37 (19.5)	96 (23.9)	-1.3 (1.1)	0.117
Surgical cases	423 (46.2)	19 (30.2)	56 (50.5)	73 (48.7)	87 (45.8)	188 (46.8)	-2.6 (1.0)	0.078
Dentofacial deformities and craniofacial abnormalities	315 (34.4)	13 (20.6)	38 (34.2)	50 (33.3)	70 (36.8)	144 (35.8)	-2.4 (0.8)	0.048
Anatomical TMJ signs	298 (32.5)	12 (19.0)	36 (32.4)	55 (36.7)	51 (26.8)	144 (35.8)	-2.4 (1.9)	0.033
Third molar evaluation	249 (27.2)	12 (19.0)	23 (20.7)	40 (26.7)	58 (30.5)	116 (28.9)	-1.6 (1.2)	0.018
Temporary anchorage devices insertion (mini-implants)	106 (11.6)	5 (7.9)	10 (9.0)	16 (10.7)	16 (8.4)	59 (14.7)	-1.5 (2.6)	0.032
Digital smile design imaging (Clear Aligner Therapy)	80 (8.7)	5 (7.9)	5 (4.5)	18 (12.0)	20 (10.5)	32 (8.0)	-1.7 (1.5)	0.023
Customized orthodontic appliance - Sure Smile^TM^	98 (10.7)	6 (9.5)	15 (13.5)	20 (13.3)	19 (10.0)	38 (9.5)	-1.1 (1.1)	0.096

** Total of respondents who answered questions about the diagnostic purpose and the Brazilian macro-region where they worked. Pearson’s Chi-square test: statistically significant results for bivariate analysis (p<0.05). *Post-hoc* Bonferroni test: different letters indicate statistically significant differences among the macro-regions with p< 0.05* and bold adjusted residuals ±2.80.

**Table 4:** Technical parameters employed in prescribing CBCT scans by orthodontic specialists in Brazil, stratified by geographic macro-regions (n=939).

Variables	Total	Brazilian macro-regions					Adjusted residual	p-value
		North	North East	Midwest	South	South East		
	n (%)	n (%)	n (%)	n (%)	n (%)	n (%)		
Which voxel size do you choose for the acquisition of CBCT images?								
I do not specify	445 (63.3)	28 (70.0)^A.B^	60 (67.4)^A.B^	82 (69.5)^A.B^	97 (70.8)^B^	178 (55.8)^A^	-3.8 (0.9)	0.006*
Small one (<0.3 mm)	59 (8.4)	0 (0.0)	5 (5.6)	5 (4.2)	9 (6.6)	40 (12.5)	-2.0 (2.6)	0.005
Large one (≥0.3 mm)	16 (2.3)	0 (0.0)	0 (0.0)	2 (1.7)	2 (1.5)	12 (3.8)	-1.5 (2.4)	0.149
Depends on the clinical application	183 (26.0)	12 (30.0)	24 (27.0)	29 (24.6)	29 (21.2)	89 (27.9)	-1.4 (1.0)	0.599
Not answered	236							
Which field of view do you prescribe?								
I do not specify	273 (38.8)	20 (50.5)^A.B^	40 (44.9)^A.B^	48 (40.7)^A.B^	66 (48.2)^B^	99 (31.0)^A^	-3.9 (1.5)	0.002*
Small area (around 4 - 6 cm)	49 (7.0)	1 (2.5)	6 (6.7)	12 (10.2)	6 (4.4)	24 (7.5)	-1.3 (1.5)	0.323
Large area (around 8 - 16 cm)	19 (2.7)	1 (2.5)	1 (1.1)	0 (0.0)	3 (2.2)	14 (4.4)	-2.0 (2.5)	0.103
Full skull	27 (3.8)	2 (5.0)	3 (3.4)	3 (2.5)	3 (2.2)	16 (5.0)	-1.1 (1.5)	0.568
Depends on the clinical application	335 (47.7)	16 (40.0)	39 (43.8)	55 (46.6)	59 (43.1)	166 (52.0)	-1.2 (2.1)	0.274
Not answered	236							

Pearson’s Chi-square test: statistically significant results for bivariate analysis (p<0.05). *Post-hoc* Bonferroni test: different letters indicate statistically significant differences among the macro-regions with p< 0.05* and bold adjusted residuals ±2.80.

Regarding the technical imaging parameters outlined in Table 4, most professionals reported not specifying the voxel size (63.3%), with the Southeast region being the least likely to adopt this practice (55.8%, p = 0.006). A small voxel size was reported in 8.4% of the sample, with a higher frequency in the Southeast region. The North and Midwest regions had the highest frequency of professionals who did not specify FOV size in their prescriptions (p = 0.006), with rates of 50.5% and 48.2%, respectively.

The variables related to the specific training of orthodontists in the use of CBCT are shown in Table 5. Among the respondents, 63.0% indicated that they had not received this training, mostly in the South region (73.7%, p = 0.014). More than half of the respondents (50.2%) received training during their specialty program in Orthodontics, with the majority receiving training approximately five years ago (40.5%). Concerning the duration of training, the majority mentioned 4-8 h (53.4%). Training courses lasting more than 20 h were more frequent in the South (23.5%) and less common in the Midwest (2.0%, p = 0.026). Only 26.5% of respondents obtained training certificates.

**Table 5:** Aspects of the training for CBCT by orthodontic specialists in Brazil, by geographic macro-regions (n=939).

Variables	Total	Brazilian macro-regions					Adjusted residual	p-value
		North	North East	Midwest	South	South East		
	n (%)	n (%)	n (%)	n (%)	n (%)	n (%)		
Have you taken any training/course for using CBCT units or images in orthodontic practice?								
Yes	260 (37.0)	11 (28.2)^A.B^	35 (39.3)^A.B^	54 (45.8)^B^	36 (26.3)^A^	124 (38.8)^A.B^	-2.9 (2.2)	0.014*
No	443 (63.0)	28 (71.8)^A.B^	54 (60.7)^A.B^	64 (54.2)^B^	101 (73.7)^A^	196 (61.3)^A.B^		
Not answered	236							
Where was this training/course in CBCT?								
During undergraduate training	2 (0.8)	0 (0)	0 (0)	1 (1.9)	0 (0)	1 (0.8)	-0.6 (1.0)	0.828
Specialty programs in orthodontics	127 (50.2)	6 (54.5)	22 (64.7)	27 (51.9)	17 (48.6)	55 (45.5)	-1.4 (1.8)	0.388
Specific CBCT course	65 (25.7)	3 (27.3)	4 (11.8)	19 (36.5)	8 (22.9)	31 (25.6)	-2.0 (2.0)	0.146
Other	59 (23.3)	2 (18.2)	8 (23.5)	5 (9.6)	10 (28.6)	34 (28.1)	-2.6 (1.7)	0.103
Not answered	686)							
When was this CBCT training/course?								
Around 1 year ago	61 (24.2)	3 (27.3)	8 (23.5)	14 (27.5)	9 (25.0)	27 (22.5)	-0.6 (0.2)	0.967
Around 5 years ago	102 (40.5)	4 (36.4)	19 (55.9)	24 (47.1)	11 (30.6)	44 (36.7)	-1.3 (2.0)	0.162
Around 10 years ago	64 (25.4)	4 (36.4)	5 (14.7)	8 (15.7)	12 (33.3)	35 (29.2)	-1.8 (1.3)	0.117
Other	25 (9.9)	0 (0.0)	2 (5.9)	5 (9.8)	4 (11.1)	14 (11.7)	-1.1 (0.9)	0.681
Not answered	687							
How long was this CBCT training/course?								
Less than 4 hours	1 (0.4)	0 (0.0)	0 (0.0)	1 (2.0)	0 (0.0)	0 (0.0)	-1.0 (2.0)	0.420
From 4 to 8 hours	133 (53.4)	5 (45.5)	22 (66.7)	30 (58.8)	20 (58.8)	56 (46.7)	-2.1 (1.6)	0.214
More than 8 to 20 hours	77 (30.9)	4 (36.4)	8 (24.2)	18 (35.3)	6 (17.6)	41 (34.2)	-1.8 (1.1)	0.320
More than 20 hours	36 (14.5)	2 (18.2)^A.B^	3 (9.1)^A.B^	1 (2.0)^B^	8 (23.5)^A^	22 (18.3)^A^	-2.8 (1.7)	0.026*
Other	2 (0.8)	0 (0.0)	0 (0.0)	1 (2.0)	0 (0.0)	1 (0.8)	-0.6 (1.0)	0.828
Not answered	690							
Was a state/province/government-recognized certificate granted?								
Yes	67 (26.5)	4 (6.3)	6 (5.4)	16 (10.7)	12 (6.3)	29 (7.2)	-0.8 (1.7)	0.015
No	186 (73.5)	7 (11.1)	28 (25.2)	36 (24.0)	24 (12.6)	91 (22.6)		
Not answered	686							

Pearson’s Chi-square test: Statistically significant results for bivariate analysis (p<0.05). *Post-hoc* Bonferroni test: different letters indicate statistically significant differences among the macro-regions, with p< 0.05* and bold adjusted residuals ±2.80.

## DISCUSSION

The findings regarding CBCT prescription by Brazilian orthodontists highlight the significant aspects of its utilization within the criteria recommended by the international guidelines for orthodontic clinical practice. The results indicate a high percentage of respondents prescribing CBCT for various orthodontic diagnoses, with slightly over 50% adhering to some form of guideline for prescribing tomographic images. The present study revealed disparities among Brazilian macro-regions concerning the criteria and diagnostic purposes for prescribing CBCT, types of guidelines used, technical aspects, reasons for refraining from prescribing the examination, and the specific training acquired. These findings may be associated with contextual factors, including socioeconomic variables, accessibility to training programs, and technological resources within the healthcare sector, particularly concerning imaging procedures. Although this study included clinicians and academic professors, most were clinicians. Future studies conducted exclusively on academics may determine whether there are differences in prescription practices between academics and clinicians.

International organizations and experts in the field have reinforced the best practice of prescribing tomographic imaging in orthodontics.^1^ ^,^ ^2^ ^,^ ^7^ ^,^ ^10^ ^,^ ^11^ This concern stems from the potential health risks of patients’ exposure to ionizing radiation in Orthodontics.^12^ Among these risks, cellular damage resulting from the alteration of DNA molecules in cell chromosomes, and consequently the development of tumors, stands out as a primary concern. Additionally, the X-radiation cytotoxicity of CBCT has been shown in oral mucosa cells, considering that cellular death and non-genotoxic mechanisms of carcinogenesis are closely related.^12^ Despite the generally low doses of ionizing radiation emitted by dental radiology, the thyroid gland is considered the region most susceptible to radiation-induced changes.^13^ ^-^ ^15^

CBCT or radiography should be performed, when necessary, for treatment planning. The biological risks associated with the routine use of computed tomography (CT) scans in orthodontics are further increased by the unique nature of the specialty, which has a high demand from young patients seeking orthodontic interventions. Orthodontics is crucial for correcting craniofacial growth anomalies during the mixed dentition and early permanent dentition phases.^16^

The stochastic effects of ionizing radiation, which accumulate over time, are particularly concerning in pediatric and adolescent patients, especially with CT scans featuring an expanded FOV, covering the entire head. These scans can result in radiation doses up to 100 times greater than those from panoramic examinations and lateral cephalometry combined.^3^ Moreover, children under the age of 10 face approximately three times more biological risk from radiation exposure than adults over 30.^2^ ^,^ ^17^ Considering the longer life expectancy of younger patients, it is imperative to prevent unnecessary tomographic prescriptions.

Guidelines for prescribing tomographic images in orthodontics should adhere to recommendations provided by esteemed institutions such as the SEDENTEXCT consortium,^2^ American Academy of Oral Radiology,^1^ and DIMITRI consortium.^7^ According to these authorities, the use of CBCT is warranted for the assessment of various clinical conditions, including patients with craniofacial syndromes and severe growth disorders, temporomandibular joint changes, orthodontic planning for impacted tooth movement, diagnostic evaluation of tooth resorption associated with impacted teeth, cases of craniofacial asymmetries, and assessment of alveolar boundaries for orthodontic interventions.

However, the findings of the present study revealed that 18.07% of Brazilian professionals did not use this vital tool to diagnose challenging orthodontic cases. Moreover, 47.4% of the professionals using CBCT did not adhere to the prescription guidelines. This outcome underscores the importance of enhancing the dissemination of global guidelines, beginning with undergraduate educational courses, to ensure appropriate and standardized utilization of CBCT in orthodontic practice. These results show the need for detailed studies on the topic in undergraduate and postgraduate courses, including standardizing the guidelines.

As excessive tomography prescription raises concerns, the underutilization of tomography is also problematic. The limited adoption rates of the AAOMR (18.6%) and SEDENTEX (2.5%) guidelines may contribute to the infrequent recommendation of CBCT for essential clinical conditions, such as cleft palate (22.5%) and craniofacial anomalies (34.4%). Cleft palate represents a complex condition requiring a meticulous orthodontic-surgical diagnosis. These findings raise concerns regarding the safety of orthodontic procedures, particularly in regions with alveolar bone deficiency or absence.

Conversely, 1% of the respondents routinely prescribed tomographic images in all or most cases. This notion of achieving optimal outcomes solely through tomographic imaging should not be accepted arbitrarily, but rather substantiated by well-designed studies, demonstrating the superiority of tomographic diagnosis over conventional imaging. Simply having a “better view” does not justify imaging with higher radiation doses, for example, for symmetric patients.^18^ Additionally, in cases like a personalized assessment of tooth movement, CBCT was recommended by 10.7% of the respondents. Currently, orthodontic aligner companies advocate tomographic examinations to visualize root positions pre- and post-treatment, aiming to prevent dehiscence and fenestration resulting from tooth movement. However, it is important to note that this recommendation lacks scientific support, as alveolar dehiscence is a common anatomical finding, affecting approximately 50% of all teeth, including those in patients with Class I malocclusion^19^ - in which periodontal changes depend on the quality of gingival tissues and the presence of associated dental biofilm,^20^ underscoring the critical role of gingival diagnosis in ensuring the safety of orthodontic treatment. 

The combination of various technical imaging parameters significantly affects the radiation dose in CT imaging. Adjusting these parameters whenever feasible aligns with the ALADAIP principle.^7^ It is imperative during prescription to specify the type of examination and provide guidance on the required FOV and voxel sizes tailored to the intended diagnostic purpose. Focusing radiation exposure solely on areas relevant to diagnosis and employing an appropriate and minimized FOV can diminish the effective dose of ionizing radiation.^21^ Similarly, using a voxel size compatible with the desired image resolution aids in radiographic protection. Considering the lack of FOV and voxel size options in CBCT prescriptions by orthodontists, a second barrier to protecting the patients may be radiological clinic protocols, working as a coadjutant safeguard. 

It is important to note that not all diagnostic purposes require high image resolution achieved through a small voxel size, which consequently entails greater radiation dose absorption.^7^ For instance, in studies concerning root resorption, the image can be optimized using a smaller voxel (high resolution) and a reduced FOV (limited exposed area). Conversely, for comprehensive head assessments required for cephalometric analysis, a medium voxel size (0.3 mm or 0.4 mm) offers adequate resolution.^22^ However, a larger FOV must encompass the patient’s facial and cranial structures. For root morphology analysis, 3D model superimposition can enhance diagnosis and provide more detailed insights into root resorption during orthodontic treatment, even with a larger FOV.^23^ ^,^ ^24^

Our study further revealed that nearly half (47.3%) of the respondents refrained from prescribing CBCT because of its high cost. In comparison, 3.6% cited the absence of a nearby radiology center in their service region. These reasons were more prevalent among specialists from the North, Northeast, and Midwest regions, which can be attributed to the lower levels of economic development in these macro-regions. In addition, the absence of a correct diagnosis when CBCT is necessary can significantly affect the orthodontic treatment plan, outcomes, and long-term stability prognosis, such as in cases of impacted teeth, oral clefts, and orthognathic surgery.^25^

CBCT prescriptions were more prevalent among younger professionals (aged 20-49) than among their older counterparts. This may be due to the evolution of educational curricula, as tomography content was not traditionally included in undergraduate or specialization courses. Due to the multiple variables influencing image formation and the biological risks posed to patients, it would be prudent for orthodontists to undergo prior training in CBCT, a step not reported by 63.0% of professionals in the present study. These findings underscore the urgent need to incorporate specific CBCT training into the postgraduate orthodontic curricula. This educational approach was reported by half of the participants (50.2%). Additionally, it is worth noting that the training courses ranged from 4 to 8 h (53.4%). Minimal training in CBCT is mandatory for specialized courses. Moreover, ongoing education should be periodically provided and regulated by appropriate councils. 

This lack of CBCT training among orthodontists has also been observed in other specialties, such as Periodontics and Oral and Maxillofacial Surgery.^26^ Given these findings, more efforts to enhance appropriate CBCT training across all dental specialties are imperative. Although general practitioners in Brazil are legally permitted to interpret images, radiologists are typically responsible for interpreting imaging studies, including identifying and reporting craniofacial anomalies. They are expected to provide a comprehensive report that includes all the relevant findings. Although orthodontists can review imaging studies, they rely on radiologists for detailed interpretations. However, they should be aware of the anomalies and follow up with a radiologist to avoid any concerns or discrepancies.

## LIMITATIONS AND RECOMMENDATIONS

This study had some limitations, particularly concerning the low number of respondents in certain Brazilian macro-regions. Nevertheless, the present results provide valuable insights into the utilization of CBCT in orthodontic practice, offering potential contributions to discussions on the appropriate and safe use of this 3D imaging modality, with direct implications for patient care. 

Based on the present findings, it is recommended that training on the use of CBCT be integrated into the undergraduate curriculum and reinforced during postgraduate and continuing education programs. This educational approach encompasses practical training and addresses specific issues regarding CBCT image prescriptions. Although no question had a specific topic concerning the knowledge of biological risks, educational training can also reinforce these risks, to avoid the indiscriminate use of ionizing radiation. These measures are essential for ensuring comprehensive and ongoing training, fostering clinical practices that align more closely with established guidelines, and enhancing the quality and safety of CBCT prescriptions by orthodontists.

## CONCLUSIONS

Most Brazilian orthodontists recommend using CBCT in clinical practice. The most common criterion for recommending CBCT was impacted teeth (74.7%), followed by surgical cases (46.2%), and root resorption (41.9%). However, less than 50% of the participants adhered to the guidelines for imaging prescriptions, and the majority (63.0%) had not undergone specific training, especially before graduation. Furthermore, there were variations among the macro-regions in several aspects of CBCT utilization. High cost played a major role in not prescribing CBCT (18.6%), mainly in the North, Northeast, and Midwest regions. These findings provide valuable insights that can inform future interventions to appropriately train orthodontic specialists, considering regional differences within the country. Such interventions are crucial for promoting standardized and evidence-based practices across diverse geographic areas.

## Supplement 1: Self-administered electronic questionnaire developed specifically for this study.

### SECTION 1

*Welcome to this scientific survey!*

The purpose of this survey is to learn about your perception of the prescription of Cone Beam Computed Tomography (CBCT) in orthodontic practice. Your opinion is important to our profession. If you agree to participate, please answer the following questions. It will take no more than 4 minutes! Your participation is very important.

1. I confirm that I have carefully read the invitation by email, voluntarily agree to participate in the survey, and I am at least 18 years old.

(_) Yes (_) No

2. What gender are you?

(_) Female (_) Male (_) Other

3. How old are you?

(_) 20 - 29 (_) 30 - 39 (_) 40 - 49 (_) 50 - 59 (_) 60 - 69 (_) 70 and older

4. Are you an orthodontist?

(_) No (_) Yes

5. How many years have you being practicing orthodontics?

(_) 5 years or less (_) 6 - 10 years (_) more than 10 years (_) more than 20 years

6. In which Brazilian state do you practice Orthodontics?

(_) Acre (_) Alagoas (_) Amapá (_) Amazonas (_) Bahia (_) Ceará (_) Distrito Federal (_) Espírito Santo (_) Goiás (_) Maranhão (_) Mato Grosso (_) Mato Grosso do Sul (_) Minas Gerais (_) Pará (_) Paraíba (_) Paraná (_) Pernambuco (_) Piauí (_) Rio de Janeiro (_) Rio Grande do Norte (_) Rio Grande do Sul (_) Rondônia (_) Roraima (_) Santa Catarina (_) São Paulo (_) Sergipe (_) Tocantins

### SECTION 2

7. Do you prescribe CBCT?

(_) No

(_) Yes, replacing 2D images

(_) Yes, as a complement to 2D images

8. Do you have a CBCT machine in your office?

(_) No (_) Yes

9. How do you use CBCT images?

(_) Evaluating all slices: axial, coronal and sagittal. (_) For 3D models analysis (_) Both

10. Do you follow any of the currently available guidelines for CBCT prescription?

(_) No (_) Yes

11. Which guideline do you follow? (check all that apply):

(_) American Academy of Oral and Maxillofacial Radiology Guidelines

(_) SEDENTEXCT Guidelines

(_) Local or national Guidelines

(_) Other (please, specify):

12. Please mark below the criteria used for prescribing CBCT in your orthodontic practice: (mark all that apply)

(_) For treatment planning, in all cases

(_) For treatment planning, in most cases

(_) During treatment, in all cases

(_) After treatment, in all cases

(_) For selected cases only (specify in the next question)

### SECTION 2

13. For what cases do you usually prescribe CBCT? (check all that apply)

(_) Impacted teeth

(_) Root resorption

(_) Dental anomalies

(_) Facial asymmetry

(_) Dental positioning

(_) Upper airway analysis

(_) Palatal cleft

(_) Surgical cases

(_) Dentofacial deformities and craniofacial anomalies

(_) TMJ signs

(_) Third molars evaluation

(_) Temporary anchorage devices insertion (mini-implants)

(_) Digital smile design imaging

(_) Personalized evaluation for tooth movement

(_) Other (please specify):

14. What voxel size do you choose for acquisition of a CBCT image?

(_) I do not specify

(_) Small (<0.3 mm)

(_) Large (≥0.3 mm)

(_) Depends on the clinical application

15. What field of view (FOV) do you recommend?

(_) I do not specify

(_) Small area (approximately 4-6 cm)

(_) Large area (approximately 8-16 cm)

(_) Full head

(_) Depends on the clinical application

### SECTION 3

16. Have you received training/course to use CBCT in your orthodontic practice?

(_) Yes (_) No

17. Where was this training/course in CBCT?

(_) During undergraduate training

(_) Specialty programs in Orthodontics

(_) Specific CBCT course

(_) Other (please, specify):

18. When was this training/course in CBCT?

(_) About 1 year ago

(_) About 5 years ago

(_) About 10 years ago

(_) Other (please, specify):

19. How long was this training/course in CBCT?

(_) 4 to 8 hours

(_) 8 to 20 hours

(_) Other (please, specify):

### SECTION 3

20. Did the training/course provide a certificate?

(_) Yes (_) No

21. Do you use a radiology specialist to interpret CBCT images?

(_) Always (_) Frequently (_) Sometimes

(_) Rarely (_) Never

22. In addition to the lack of indication, is there any other reason that prevents you from requesting a CBCT? (check all that apply)

(_) High cost

(_) Not having a specialized radiology center nearby

(_) I do not believe CBCT brings new information

(_) Radiation doses (_) No specific reason

23. When you refer patients for CBCT, do you inform them about the radiation dose, compared to conventional imaging?

(_) Always (_) Sometimes (_) Never

24. Please share any other considerations you may have regarding the prescription of CBCT in orthodontic practice.

Thanks for your participation!

Now just click on “done”, below.
